# Exploring the Impact of the First Wave of COVID-19 on Social Work Practice: A Qualitative Study in England, UK

**DOI:** 10.1093/bjsw/bcab166

**Published:** 2021-08-17

**Authors:** Tom Kingstone, Paul Campbell, Alina Andras, Karen Nixon, Christian Mallen, Lisa Dikomitis, Q-COVID-19 Group, Alina Andras, Paul Campbell, Lisa Dikomitis, Toby Helliwell, Tom Kingstone, Christian Mallen, Kay Polidano, Michelle Robinson, Thomas Shepherd, Brianne Wenning

**Affiliations:** 1 School of Medicine, Keele University, Staffordshire ST5 5BG, UK; 2 Research and Innovation Department, Midlands Partnership NHS Foundation Trust, St George’s Hospital, Stafford ST16 3SR, UK; 3 Social Work Learning Academy, Adult Social Care, Midlands Partnership NHS Foundation Trust, The Coach House, St Michaels Court, Lichfield WS11 6EF, UK

**Keywords:** interviews, pandemic, public health, qualitative research, remote working, resilience

## Abstract

The COVID-19 pandemic signalled a radical shift in health and social care services globally. In UK, many of the people with existing social care needs were identified as ‘clinically vulnerable’ to COVID-19. Those at greatest risk were encouraged to adhere to additional public health measures that inadvertently exacerbated social disadvantages. Social workers were challenged to ‘dig deep’ to continue to provide services as usual. However, problems implementing new ways of working were reported but not examined in-depth through research. Our study explored experiences and perceptions of social workers responding to the first wave (April–July 2020) of COVID-19, in England, UK. Interviews with thirteen social workers, all working in the West Midlands region, were conducted via telephone or online video. Transcripts were analysed using reflexive thematic analysis. We use ‘managing uncertainty’ as a central concept underpinning the four themes identified after analysis: (1) providing social care at a physical distance, (2) negotiating home/work boundaries, (3) managing emerging risks and (4) long-term implications for social work. We discuss our findings in the context of resilience and organisational adaptation. Social workers in our study demonstrated resilience in action and rapid adaptation to new practices, but equally expressed concern about short-term efficiencies being prioritised over individual service user needs.

## Teaser

The COVID-19 pandemic signalled a dramatic shift in how health and social care services were delivered to members of the public. Social workers reported challenges implementing new ways of working during the first wave of COVID-19. However, such challenges received little coverage in the media or in research. This article explores the views and experiences of social workers responding to the first COVID-19 wave in England, UK (April–July 2020). Telephone and online interviews were carried out with thirteen social workers active in the West Midlands region. Social workers shared experiences of how they managed uncertainty—a core theme in the study. Interviewees described managing the uncertainty of: (1) providing social care at a physical distance, (2) negotiating home/work boundaries, (3) managing emerging risks and (4) contemplating the future of social work practice. Our study demonstrates how social work professionals adapted rapidly to new practices during the first wave. However, social workers raised concerns about the impact of short-term solutions on long-term needs of service users. Implications for research and practice are discussed with reference to key literature.

## Introduction

The 2020 pandemic caused by Severe Acute Respiratory Syndrome Coronavirus (SARS-CoV-2) most commonly termed ‘COVID-19’ dramatically and suddenly impacted on societies across the globe.

At the time of this study, public health legislation in England had been introduced to protect the population that distinguished categories of clinical vulnerability—the risk of experiencing COVID-related complications (NHS, 2020). People defined as high-risk, included those undergoing targeted cancer treatment and people taking high dose steroids, were advised to ‘shield’ by not leaving their home and by avoiding unnecessary contact. People defined as moderate risk included individuals, for example over the age of seventy years, with severe asthma, heart disease, diabetes and/or are pregnant were advised to remain vigilant and strictly maintain physical distances from people outside their household. COVID-19, and the implemented public health measures, inadvertently exacerbated existing vulnerabilities (Miller and Lee, 2020) and inequalities derived from ethnicity, age and economic deprivation (Bowleg, 2020). Many of the people defined as high risk who were shielding had existing social care needs (Comas-Herrera *et al.*, 2020). Mortality rates evidenced a disproportionate impact of COVID-19 on care home residents (Gordon *et al.*, 2020), signalling a failure in the UK’s response to COVID-19 (Lewis, 2020). Through physical distancing and shielding, older adults lost connection with crucial support networks and services (Berg-Weger and Morley, 2020).

The COVID-19 pandemic has led to a major transformation of health and social care in England, both for those at the receiving end of care and those who provide care. However, research examining challenges and transitions faced by social workers has largely been overshadowed by those faced by healthcare professionals. The status of social workers as essential or ‘key workers’ was also contested (Lipe, 2020); clear examples of the dominant biomedical paradigm. Whilst survey data, commentaries and reflective essays have done much to raise the profile of challenges experienced in social work, a lack of scientific rigour and qualitative depth remain evident. An international survey examined social worker perspectives during the COVID-19 response (Banks, 2020). Survey respondents indicated ethical concerns relating to establishing remote relationships with service users, and risks opposing trust, privacy, dignity and service user autonomy. Golightley and Holloway (2020a) discuss matters of maintaining set physical distances and using personal protective equipment (PPE). In particular, how these impact social work routines and efforts to nurture trusting relationships with clients. During the early stages of the crisis, social workers were asked to ‘dig deep’ to adapt and make use of emerging resources (e.g. digital technology) and to capitalise on reductions in bureaucracy to support vulnerable members of society (Golightley and Holloway, 2020b). It is not known how such resources have been perceived and utilised. To date, the experiences of social workers and transformations in service provision during and because of the COVID-19 pandemic have not been explored in research.

The aim of this research was to explore social workers’ perspectives on the impact of the COVID-19 pandemic on social work practices in a UK context. Findings represent views from the first wave of the pandemic (Spring 2020) and are expected to add to the COVID-19 qualitative research evidence base. Study findings will increase our understanding of the pandemic’s impact and sequelae on social work practice.

## Methods

### Study design

A qualitative research approach using semi-structured interviews with social workers to explore their perceptions, experiences and understanding of the COVID-19 pandemic and the public health measures. Such qualitative study design allowed us to collect in-depth views from social workers directly involved in this new and dynamic situation. This research was undertaken by a multidisciplinary research team comprising a social scientist (T.K.), a psychologist (P.C.), a health service researcher (A.A.), a social worker (K.N.), a general practitioner (C.M.) and a social anthropologist (L.D.).

### Setting and participants

This research was conducted during the first wave of the COVID-19 pandemic in the UK, between April and July 2020, when both researchers and research participants lived and worked under public health measures.

Active social workers, working in the West Midlands region (England, UK), were recruited via snowball methods through the research team’s existing professional networks (Bowling, 2014). A snowball sampling strategy was applied in order to effectively utilise the extensive professional networks of the study team. Study information was distributed via senior representatives from local authorities, NHS Trusts and third sector organisations. Social workers were invited to express their interest in participating to the research team and ask any questions they may have. If they agreed to participate, they were asked to complete an online consent form. Once e-consent had been received, a member of the research team contacted the social worker to arrange a suitable time and format for the interview. Participating social workers distributed study information to their colleagues to support ongoing sampling.

### Data collection

One-to-one semi-structured interviews supported the exploration of this previously under-examined topic. Interviews were conducted by experienced researchers (T.K. and P.C.). Interviews were conducted remotely by telephone or MS Teams video call, depending on interviewee preference. An interview topic guide (Supplementary File S1) was developed with input from social work stakeholders. The guide covered the following broad topic areas with prompts to facilitate conversation: demographic characteristics (age, gender, occupation and years of experience), challenges arising from COVID-19, knowledge about COVID-19, remote working, supporting service-user needs, personal safety and wellbeing and long-term implications. T.K. and P.C. met regularly to review prompts within the topic guide as an iterative process in parallel with data analysis.

### Analysis

Interviews were, with participant’s consent, audio-recorded and transcribed *ad verbatim* by an external transcription service. Data analysis followed a reflexive thematic approach using principles of constant comparison to analyse across different cases (Fram, 2013; Braun and Clarke, 2019). The lead author (T.K.) coded all transcripts, noted down interpretations and identified preliminary themes. The research team, representing a mix of different backgrounds, coded a sub-set of transcripts, discussed interpretations with the lead author and agreed a final set of themes. We applied the concept of saturation: a decision to cease data collection was taken once the team felt confident that saturation, at a thematic level, had been achieved (Saunders *et al.*, 2018).

### Ethics

The study gained ethical approval from Keele University Faculty of Medicine and Health Sciences Research Ethics Committee on 21 April 2020 (Ref: MH-200123).

## Results

In-depth interviews were conducted with thirteen social workers. Characteristics of the participants are presented in Table 1. Ten participants were female and three were male; average age of forty-two years (range: twenty-nine–fifty-five years). Social worker roles were distributed across levels of seniority (six social workers, two senior social workers, two principal social workers, one consultant, one consultant educator and one team manager). Participants covered a range of urban and/or rural localities and populations.

**Table 1.** Participant characteristics

Participant ID	Age	Gender	Role	Years in service
SW01	29	Female	Consultant Social Work Educator	8
SW02	49	Female	Principal Social Worker (Adult Mental Health)	30
SW03	33	Female	Social Worker (Child Mental Health)	7
SW04	43	Female	Team Manager (Third Sector)	19
SW05	55	Female	Consultant Social Worker (Adult Social Care)	32
SW06	51	Female	Principal Social Worker (Older Adults and Disability)	25
SW07	45	Male	Social Worker (Adult Social Care)	8
SW08	43	Female	Social Worker (Adult Social Care)	14
SW09	39	Male	Senior Social Worker (Child Protection)	7
SW10	52	Female	Senior Social Worker (Child Protection)	6
SW11	42	Male	Social Worker (Adult Social Care)	6
SW12	31	Female	Social Worker (Child Protection)	<1
SW13	40	Female	Social Worker (Adult Social Care)	7

Interviews were conducted via MS Teams video call (*n *=* *3) or telephone (*n *=* *10) and lasted on average 57 min (range: 32–72 min). Data are labelled using unique participant identifiers to maintain anonymity (e.g. SW01).

Through our analysis, we identified a central overarching theme: ‘managing uncertainty’. This underpins each of the four main themes: (1) Providing social care at a physical distance, (2) negotiating home/work boundaries, (3) managing emerging risks and (4) long-term implications for social work. The central theme helps to reconcile some of the complexity in participants’ attempts to make sense of their role and activities during the pandemic.

## Providing social care at a physical distance

Participants described remote working as a key challenge arising from public health measures during the pandemic. Remote working constituted a shift in routine practices and required radical changes to service delivery, increased reliance on digital technology and greater extent of lone working. Each issue is elaborated below with participant data.

### Compromising on quality of service provision

Participants described being determined to maintain ‘business as usual’ in line with core social work principles and values. However, they accepted that COVID-19 restrictions impacted standard practice and the overall quality of service provision:

Because of the nature of the job that we do, it doesn’t sit comfortable with me that we’re not doing it in a direct face-to-face way and I think that there has to be some impact in terms of the quality. (SW11)

An increased reliance on telephone communication with service users was identified as a barrier to fundamental practices, including relationship building:

I’ve always thought of myself as being good at doing relationships, social work. You know, you build a rapport with a client and I haven’t been able to do that to the same extent and actually like, I’m getting to a point with one of my clients, where I’m going to have to say, if you don’t want my help, that’s fine. No one’s forcing you all right and thank you very much for engaging thus far. But usually I’m the person who gets in every single door, who never has any problem with that kind of thing. So, I guess there is an impact on my ability to develop relationships with clients. I do seem to end my assessments at the moment not feeling like I’ve been able to build the rapport that I would have wanted to. (SW09)

Concerns were raised that changes in practice and the format of contact meant the needs of some service users were being overlooked:

Some of the people aren’t getting the service that they necessarily need because some of those children and young people need that face-to-face support and they aren’t getting that. And that could be for lots of different reasons like family dynamics. I think that’s the biggest one. Family dynamics are a barrier towards accessing the child at home. And school was the safe place where we could access them. (SW03)

Some participants in adult social care settings still conducted face-to-face visits with service users, where social restrictions permitted. For example, one participant described meeting with a service user in their garden. However, such face-to-face meetings were rare during the first wave of the COVID-19 pandemic. The threshold for these visits had been raised, as one participant pointed out:

Often, we will just pop out just to check they’re all right. At the minute, you’re trying to avoid at all cost going out, which is not natural, actually, for a social worker, but … Also, you’re trying to balance the safety of the staff as well, and it’s tricky. (SW08)

Remote working also inadvertently reinforced inequalities amongst service users, particularly in relation to material resources, as one participant described:

And then, one of the issues actually is getting resources to children to use. So, the expectation that kids have got laptops, computers, phones that they can call us. Some of the children that we work with don’t. Printers, so, the resources, they’re not getting the resources and stuff like that. So, that’s hard. So, we have to be creative in that. (SW03)

Compromises on services were understood to be short-term and accepted amongst service users and providers:

It’s about explaining that, at the moment, this is how it works, but we will have to revisit after COVID-19 in a lot of situations. It’s having to accept that, but I think most people have compromised. People that would normally ask for face-to-face have compromised themselves. I think that they’ve said, don’t worry; we’ll see what we can do in the meantime. (SW05)

### Going digital

Participants described different levels of engagement with digital technology. In theory, digital technology should enable social workers to overcome physical barriers imposed by restricted face-to-face contact with service users, colleagues and external partners. In practice, adoption of digital technology was challenging. Our interviewees experienced difficulties when specific technology was suddenly no longer supported and/or replaced:

They gave us the kit. They gave us headphones. They put all these Teams, Skype and WhatsApp on our phones. Jabber. What they didn’t do was give me instructions on how to use it. I’m not very good at IT. I can now use Teams. I haven’t got my head around Jabber. They took Skype off me and I knew how to use Skype. (SW11)

The adoption of digital technology by social workers in this study required a period of rapid learning. Participants shared frustrations with functionality:

The usual way of doing it, is we sit around a big table at a central area, looking at boards and discussing the risks and protective factors and they have, those meetings have still gone ahead. One person’s in a meeting room, one person’s on a video call, one person’s on a conference call but everyone is still contributing into a meeting of sorts. I mean just the most, I did some early on, and I would have to have one mobile in my hand on speaker phone, another mobile with a different professional on speaker phone, one person on Jabber, one person on Teams or whatever and all of us trying to talk. It was horrendous and the family came away saying, I didn’t hear most of that. So that was very challenging and oppressive and all the things you don’t want those meetings to be. (SW09)

Participants were clear about the limitations of utilising digital technology to work remotely with clients; many feared missing important factors or cues due to their partial view of a service user’s reality:

I think the challenge has been … working from home for most of this period and doing a lot assessments using phone calls or using Microsoft Teams or Skype, which in some cases is okay, but in others, for example, where there’s an aspect such as domestic abuse present, I think it’s quite worrying that you may potentially be speaking to a victim of domestic abuse and trying to get an understanding around that person’s relationship, but you're missing all the body language and cues. And you don’t really know if the perpetrator of the domestic abuse is present during that conversation and therefore the information they provide you may be limited due to their environment. (SW11)

Interviewees also highlighted the advantages of using technology with service users and families:

I love that when we’ve been able to share the screen and do collaborative work with the kids. Like, we’ve done … I’ve just done really good pieces of work with them where we’ve both then put it on, say, a worksheet and filled it in together. And then, I’ve sent them those resources by email and that’s been really useful for them because that’s probably … I wouldn’t do that normally if I was seeing them at school. Or we would write it down, or I’d take it away or take a picture and give it to them, it’d probably get screwed up in the bag. So, actually, some of it’s really good. Yes, and some of it’s been good because their parents have got involved, whereas we wouldn’t normally always see them. So, they’ve sat in on some of the sessions and contributed or used some of the stuff that we’ve told them, techniques or interventions and things about mental health. (SW03)

It was felt that digital technology had a place in social care but as an addition to routine practice and not as a replacement:

I think that people see it as an interim at the moment, anyway, so it’s not like this is going to be it forever, which I think is why people are tolerating the virtual contact. And, obviously, they can see it happening all over the world, so this is a shared isolation that everybody’s having to experience. But I think people, hopefully, have the hope that this is going to end at some point, things are going to get back to some resemblance of normality, although probably not as quick as people may anticipate. So I think there is a place for virtual contact, but not to replace [face-to-face]. (SW02)

### Lone working practice

Lone working practices contributed to growing feelings of isolation amongst social workers in this study, which seemed to impact practitioners and managers. Working at a physical distance from colleagues interfered with communication and support networks:

Conversations within the team about cases and bouncing off each other. And I mean, we can email, but it’s not the same as just having a quick chat with someone once you come off the telephone. So, you do feel a lot more isolated than previously, when you were in the office environment. (SW05)

Lone working appeared to impact at service levels with operations becoming more siloed:

We’re all in a silo working on our own, no one knows what cases each other are working and so we haven’t called on each other to help out for risky visits and things like that. So, that hasn’t felt great and at times you’ve felt quite alone in your decision making in that it can be hard to speak to managers and we don’t have a good system in (Town X) for senior, no one speaks to the senior. (SW09)

## Negotiating home/work boundaries

The majority of participants had been working from home exclusively, since the COVID-19 lockdown started. A minority continued to conduct face-to-face visits with service users (albeit limited in number) and/or continued to work in their usual office environments, observing strict physical distancing guidelines. Working from home provided a number of benefits but relationships between participants and their home-space required renegotiation; which was problematic in a social work context.

### Working flexibly

Participants appreciated not commuting to and from work on a daily basis; time spent travelling to and from appointments with service users and external partners was also reduced. Social workers in this study shared views on how this enabled them to work more flexibly:

Remote working has been fine, and reduced travelling time, which is always a challenge when I’m having to drive to >Place W< which is forty minutes there and forty minutes back. So that’s substantial amount of time saved that I could be working, and I’m probably working more being at home. But I guess it’s having the flexibility to be able to manage that, which is something that most people are doing, working more flexibly in relation to hours, perhaps not starting till ten and finishing at six, or working in the evenings and stuff like that. That’s been easier. (SW02)

The ability to capitalise on flexible working had positive implications for work/life balance.

### Competing home/work expectations

Study participants described being required to log-in to online systems (e.g. Microsoft Teams) to provide a visible presence: to be seen to be available and ‘at work’. However, in having to maintain an online presence, social workers in this study perceived a level of pressure and expectation to be visible at their desks throughout the day:

If I was at work normally, I would not be sitting at my desk nine to five during the day. I’d be going, it might be the co-op for my lunch or I’d be going out to visit, so, I’d be in the car and all those things that you do normally in the day that I’m not doing now. So, you do feel like you’re a bit under a microscope. (SW03)

Household composition had a major impact on the ability of participants to maintain new routines. Some had young children who required home-schooling; others were sharing their home with partners who were shielding due to health conditions. These additional responsibilities added further expectations and demands:

Having older kids at home, trying to work. You’ve got this expectation, and then worrying that the kids are not doing enough work. Because I’ve got four kids in different years at school, and trying to educate four kids and do social work just … It got on top of me at one point, but now I think I’m just trying to think, as long as they’re safe, they’re just going to have to catch up if they do get a bit behind. So, yes, I’m a bit more settled with the whole situation at the minute, but I did get at one point really stressed and down about all of that. (SW08)

The perceived expectation to be seen to be working was exacerbated by household situations in which competing responsibilities were present.

### Protecting personal space

The use of digital technology to support home working permitted service user entry, albeit virtually, to social worker’s home space. Participants shared contrasting views; for some, this level of access was undesirable:

I said to my manager I can’t deal with this man in my house. I don’t have people swearing and shouting at me in my house. I don’t have people draining me in my house. I’m sorry, I’m not going to call him from my house. To be fair, my manager took on that role and he called this father and he was the one doing email contact with him for me. To be fair, that protected me, but, again, that’s my boundary, but my manager stepped in and protected me from that, so to be fair he was really good. (SW10)

Concerns were raised about how certain aspects of home life were not for public view:

I never gave my colleagues permission to come into my house but now we’re having virtual team meetings, and everyone can see my dirty laundry drying on the airer behind me. And that kind of thing. And that’s why we do video calls but when you do a video call with someone, you can see in their house, but they can see in yours and I felt very conscious about that. And said all right, we’ve seen each now and I’ve seen the bits of the house I need so I’ll just end the video call and we’ll just speak on the telephone, so you can’t see in my house because I don’t really want you in my house. Which is kind of oppressive. (SW09)

In contrast, some social workers in this study described the ability to share more of their personal lives with service users useful for rapport. For instance, two-way video calls afforded service users the opportunity to enter into the worlds of social workers, which helped breakdown hierarchies between social workers and clients:

One young person asked to see my dogs and stuff. And I don’t know if they feel like there’s a bit more human side to you sometimes, because you’re at home. I was with a woman and her son the other day and they were laughing because they could hear one of my kids was doing something downstairs, shouting or playing or on the game or something. They were laughing, and I think it’s that kind of element of everyone’s going through the same thing, that we’re just normal. (SW03)

## Managing emerging risks

Risk management and assessment are commonplace in standard social work practice. COVID-19 however generated a new set of risks and procedures to be implemented, information around which seemed vague at the outset. Novel situations were considered as part of risk assessment processes; the use of PPE was described as an example.

### Assessing risk in context of COVID-19

Risks associated with COVID-19 infection created a challenge for social workers in this study, particularly for the minority conducting face-to-face contact with service users. In these circumstances, social workers in our study were forced to confront the notion that they themselves posed a risk to service users:

This is the first time that the risk coming to a family from us could be grandma gets very poorly and dies because we didn’t realise we were asymptomatic. We’ve had to think very carefully about is this visit needed? (SW09)

Interviewees explained they balanced the risks associated with COVID-19 infection against the needs of service users. One participant described a situation involving a mother seeking refuge for herself and five children. The social worker broke physical distancing restrictions in order to provide support:

I was very aware of the risk. Equally, what do you do? Do you leave the children and the mum in a very risky situation, or, do you kit yourself up and you just do it. I will always kit myself and do it, I’m afraid. I can’t leave children in that situation if I can be part of the solution to it. (SW10)

This participant’s reference to ‘kit’ denotes PPE (covered below). Social workers in this study acknowledged that risks were shared between themselves and service users; this interdependence relied on both parties being open and honest about their health and potential symptoms:

Yes, one of my colleagues also, I was working with the mother and she has tested positive and they have been coming and going from the property and she’s not let on that has been. So, we are pretty much led and it’s about working openly and honestly and the risk implications, I would rather be working from home if it means I’m going to put myself at risk or put my children and wife at risk. Like you said, it’s very much led by the service users being transparent. (SW11)

### Accessing PPE

The majority of participants described having adequate access to PPE (at the time of interview); however, confusion over procedures was described. The use of PPE with service users, particularly those with cognitive impairment, was considered to further compromise ‘natural’ approaches to social work:

If we do visits, even if there’s no COVID symptoms, everyone, I think, as far as I’m aware, I think everyone that’s gone out is wearing full PPE, and we have to take all precautions anyway. So, it’s not your natural social work approach. I mean, we’re normally quite casual and friendly, not wearing masks and things, so it’s quite different. And I think especially someone with dementia, it’s quite intimidating, really. But unfortunately, we have to safeguard ourselves as well. So, yes, that is the main difference, is that we have to wear protective equipment. (SW08)

Continuing challenges were described by those participants who were seldom required to apply PPE:

I haven’t had to use PPE at all. I’m picking some up this afternoon to take to [Town Y] tomorrow because I live in [Town Z] so this is somewhat from [Town Z] place at the county council. I’m going to fetch some of that later, but I don’t even know how to use it. I think there were some concerns at the beginning, but there seems to be a lot more PPE now than we had. (SW05)

## Long-term implications for social work

Social workers in this study reflected on the potential long-term impacts of COVID-19. Participants shared concerns about hidden needs of vulnerable members of society, increased demand, financial cuts but also opportunities to take forward new ways of working. All participants described experiencing an initial reduction in the demand for social work services at the start of lockdown:

In the beginning, it went very quiet. We never used the ‘Q’ word in social care, but we’ve been able to use it quite liberally. Yes, it did become very quiet for two reasons. One reason is that people didn’t want to come to us because they didn’t want to have that contact or have to have people. The reliability of informal care in families was a lot better, I think; and secondly because of the COVID-19 bill. The easement of that bill into adult social care within this area. I think that then stopped a lot of less urgent work coming through to us. (SW05)

This initial reduction caused many to anticipate a future upsurge in demand:

Caseloads have not exceeded at all; they have not risen. There’s a steady turnover, but it’s sort of business as usual, really. People are responding to urgent situations, as we would do anyway. And it’s surprising that we’re not getting that surge of work that I think was anticipated, whether this is, and we’ve said this for ages as well, whether this is the calm before the storm. (SW02)

Participants shared concerns about potential financial impacts of COVID-19, these concerns were often based on past experience in times of austerity and described with reference to implications for vulnerable members of society:

The government has provided funding … There’s still going to be a deficit to the local authority for the amount of support that they’ve had to put in. So, again, I feel like we’re always cutting and cutting, and I think it’s just going to mean we’re still going to have to be looking at cuts. And just there’s going to be more cuts, which means less services to the people. And then not promoting wellbeing, in my point of view, it’s just making things harder for vulnerable people in the society. (SW08)

The pandemic has provided opportunities for social workers to update practices; this has resulted in economic efficiencies for services (e.g. reduced travel expenses) and enhanced flexibility for staff. Study participants also appreciated new levels of trust and autonomy:

I’ve worked in an authority where a service delivery manager didn’t trust any of the social workers and wanted to see them working at all times. And this thing about we are trusted to do all our work at home and check emails and manage our own time and our diaries and things like this. I think that’s a fantastic thing and hopefully that extends I think it probably will. (SW09)

However, concerns were raised about the extent to which social work should operate remotely in the future and what consequences this held for service users:

I think in relation to longer-term impact, I think it’s perhaps anxieties that this virtual working is going to be perhaps taken forward in the future. I certainly think we will be changing the way we work, absolutely. If it will save time to be able to focus on work more, that’s great, but not at the expense of vulnerable people in the community. (SW02)

## Discussion

### Summary of findings

Our research explored social workers’ perspectives on the impact of the COVID-19 pandemic on social work practices in a UK context. Findings show how social workers in this study responded to the challenges presented by COVID-19 public health measures and how they remained determined to deliver ‘business as usual’ despite changes in practice. Rapid change and adaptation were described in relation to remote working and delivering social work at a physical distance. Compromises on service quality were described as were concerns about the weakening of team cohesion and inter-agency working. Study participants experienced advantaged and disadvantages in the use of digital technology in remote working. Working from home, although convenient for many, forced individuals to renegotiate the relationships they shared with home-spaces and with others occupying those spaces (whether in person or virtually). COVID-19 presented novel risks for social workers to actively assess and manage; for the first time, they confronted the notion that they themselves posed a risk to service users. In looking to the future, participants’ views seemed partially distorted by the perceived threat of impending financial cuts, loss of services and anticipated surge in demand. Fears about which seemed to have been normalised through recent experiences of austerity. Social workers in this study did not reflect on what cuts may mean to them personally, but on the implications for vulnerable members of society. To deliver social work in the face of such adversity has been a show of resilience-in-action.

### Comparison with literature

Social workers were challenged to ‘dig deep’ during the 2020 pandemic (Golightley and Holloway, 2020b). Our research provides authentic accounts of transformation and resilience amongst a group of social workers operating in uncertain times with rapidly changing information, advice and practices. Participating social workers described compromises on services which challenged their core values and principles. For example, providing social work from a physical distance via telephone and video technology challenged relationships between people (service users, peers and managers) and places (home, work). Boundaries often blurred and concepts of *trust* (between service users and practitioners, practitioners and senior management), *reciprocity* (sharing of home environments with service users and peers) and *ethics* (considering what is ‘the right thing to do’ within the circumstances of a pandemic) were paramount. The findings from this research add qualitative evidence to published survey findings from Banks (2020). Working from home challenged social workers in this study to maintain online visibility to others; participants described feeling under greater levels of scrutiny, which risks burnout in the long-term (Peinado and Anderson, 2020).

Resilience is embedded within social work education and training and developed through reflective practice; being prepared to manage adversity has become synonymous with the occupation (Cleveland *et al*., 2019; Banks, 2020). Post-structuralist perspectives on resilience provide a critical view on the positioning of social workers in relation to power and the empowerment of service users (Prowell, 2019). Applying this post-structuralist lens on our findings reveals how, in relation to resilience, social workers in this study established new remote working practices during the pandemic that inadvertently reinforced material inequalities experienced by service users (e.g. access to digital technology resources and capabilities). Positive examples of empowerment and anti-oppressive practices were described in relation to enhanced opportunities for creative and collaborative activities with service users. However, participating social workers themselves raised concerns about vulnerable members of society being ‘left behind’ and lacking power should new remote working practices continue beyond the pandemic.

Meyer (1982) theorised phases of adaptation that organisations traverse in response to environmental jolts, defined as sudden and unprecedented events. Bryce *et al.* (2020) applied Meyer’s model to the NHS during the COVID-19 pandemic to understand issues of resilience and uncertainty. We discuss implications of this model in relation to findings from a social work context. Meyer (1982) described three phases of adaptation: *anticipatory* (preparations that occur before the jolt), *responsive* (activities that take place during the jolt) and *readjustment* (events that occur in the aftermath). Meyer’s (1982) responsive phase seems most pertinent to the current study and the experiences of participating social workers; limited anticipatory activities were described. Meyer (1982) recognised how organisational ideologies shape reactions to jolts, which may in themselves be constrained by structures. Participating social workers depicted social work as a profession used to working in times of adversity; thus, COVID-19 was framed as yet another challenge to the service. In practice, social workers in this study described compromising on individual ideology (core values and beliefs about what constitutes ‘good’ social work) when rapidly shifting to new online delivery practices; this generated tensions with concerns raised about reduction in service quality and increased likelihood of missing cues. Thus, imposed structures (e.g. public health measures, drive for economic efficiency borne about from austerity) worked to shape responses to the pandemic capitalising on social worker’s propensity to work during time of adversity.

Meyer (1982) argues that during the stage of readjustment: ‘organizations’ resiliencies are revealed, and the consequences of their adaptations can be assessed’ (p. 531). The British Association of Social Work (BASW) predicted positive outcomes for services in the UK post-COVID-19 including an enhanced public perception of social work, closer working with mental health services, greater clarity on social work values and improved working conditions (Allen, 2020). However, the outcomes remain unclear at this point and it remains to be seen how social work rebounds in the aftermath of the pandemic. Descriptions from participating social workers imply a reactionary approach to the pandemic, which is to be expected given the early stages of the pandemic; however, this approach is likely to be sub-optimal in the long-term and should be continually reviewed (Bryce *et al.*, 2020). Social workers in this study shared a good deal of pessimism about impending financial cuts and what this will mean for service provision and vulnerable members of society—a symptom of historical cuts and challenges.

### Strengths and limitations

This study provides new qualitative insights into social work practices from one English region (West Midlands) during the first wave of the COVID-19 pandemic. Interviews were conducted with a diverse range of social work professionals (level of seniority, gender, age, years in service) that reflects the wider social care workforce (Skills for Care, 2021). Data analysis was performed by a mixed team representing different disciplinary backgrounds, which made for robust discussion and development of themes. The sample size, depth and quality of the data supported saturation at a thematic level.

Several limitations of the study are noted. Findings may have limited transferability to other UK or international contexts. Analytic themes were selected to tell a coherent story based on participant shared experiences; data analysis revealed topics not reported here (e.g. information management, public perception of social workers and strategies to maintain own wellbeing). Further research could examine the impact of the COVID-19 pandemic in different geographical contexts and with specific demographic groups, such as social workers from ethnically marginalised backgrounds or from those who work with people who lack capacity, to achieve greater granularity. The timing of data collection (April–July 2020) meant experiences and views on practices relate to the early stages of the pandemic only; further longitudinal follow-up should examine longer-term changes. Further research should also consider service-user views to understand their experiences of remote services and perceptions of boundaries, risk, trust and reciprocity.

## Conclusions

Short-term compromises on services are accepted by service providers and users. However, should the pandemic continue and should compromises turn into the ‘new norm’ for social work then acceptance may turn into frustration. The integration of digital technology has been positive for some but not all; further training is required to optimise engagement and use with service users. Observing how social work readjusts in the aftermath of the pandemic will be of interest; what lessons will be learned and taken forward? To what extent will services revert back to pre-COVID-19 activities and standards? Social workers are predicting an upsurge in demand in the aftermath of the pandemic; population trends in terms of increased unemployment rates, incidents of domestic violence, number of children living now living in poverty in the UK and emerging evidence on the impact of ‘long-COVID’ (persistent and often debilitating symptoms following COVID-19 infection) (Kingstone *et al.*, 2020) increase the likelihood of a surge. Social work leaders need to balance efficiency facilitated by new technology against the needs of social workers to sustain core values and principles, otherwise vulnerable people risk being left behind.

## Supplementary Material

bcab166_Supplementary_Data
