# Supplementary material for: Exploring the Impact of the First Wave of COVID-19 on Social Work Practice: A Qualitative Study in England, UK
Source: Br J Soc Work. 2021 Aug 17:bcab166. doi: 10.1093/bjsw/bcab166 (PMC8436403; doi:10.1093/bjsw/bcab166)
Supplement: bcab166_Supplementary_Data [file bcab166_Supplementary_Data.docx]

**INTERVIEW TOPIC GUIDE FOR SOCIAL WORK PROFESSIONALS**

**This document has been adapted from the original for the purposes of publication*

1. **Background information about participant**

- Gender, age, occupation, social work setting/context, years of experience, living situation (living alone/ with family)

1. **Challenges arising from COVID-19**

- Main challenges since the outbreak of COVID-19 in the UK (Probe on each challenge)
- Interpretation of the term ‘risk’ with regards to COVID-19
- Experience, preparedness and prior training for pandemic crisis.

1. **COVID-19-related knowledge**

- Communication from health and social care authorities
- COVID-19 training
- Preparation and confidence in working with service users with COVID-19
- Current role and responsibilities

1. **Working remotely with service users**

- Experience of remote consultations and remote social care ‘visits’ so far
- Previous experience of video and telephone consultations
- Perceived of remote consultations by service users
- Benefits of remote consultations
- Challenges of remote consultations

1. **Non-COVID-19 illness/social care support and needs**

- Impact of COVID-19 on management of non-Covid19-related cases / issues
- Change in demand for care since start of pandemic

1. **Personal safety and wellbeing**

- Main concerns during the pandemic
- Access to adequate personal protective equipment (PPE)
- Negotiating work and family responsibilities
- Reaction to you in your role from others during this pandemic
- Activities to support your physical and mental health
- Accounts of negative behavior/animosity/violence/abuse from the public

1. **Implications for primary / social care beyond epidemic**

- Impact on service users in the future after COVID 19
- Opportunities/lessons to be learned from this situation

1. **Is there anything you would like to add?**
